# Resting State BOLD Variability Is Linked to White Matter Vascular Burden in Healthy Aging but Not in Older Adults With Subjective Cognitive Decline

**DOI:** 10.3389/fnhum.2019.00429

**Published:** 2019-12-05

**Authors:** Vanessa Scarapicchia, Mauricio Garcia-Barrera, Stuart MacDonald, Jodie R. Gawryluk

**Affiliations:** ^1^Department Psychology, University of Victoria, Victoria, BC, Canada; ^2^Institute on Aging and Lifelong Health, University of Victoria, Victoria, BC, Canada; ^3^Division of Medical Sciences, University of Victoria, Victoria, BC, Canada

**Keywords:** signal variability, Alzheimer’s disease, subjective cognitive decline, cerebrovascular health, aging, biomarkers, white matter

## Abstract

**Background**: Alzheimer’s disease (AD) is the leading cause of dementia. A lack of curative treatments and a rapidly aging global population have amplified the need for early biomarkers of the disease process. Recent advances suggest that subjective cognitive decline (SCD) may be one of the earliest symptomatic markers of the AD cascade. Previous studies have identified changes in variability in the blood-oxygen-level-dependent (BOLD) signal in patients with AD, with a possible association between BOLD variability and cerebrovascular factors in the aging brain. The objective of the current study was to determine whether changes in BOLD variability can be identified in individuals with SCD, and whether this signal may be associated with markers of cerebrovascular integrity in SCD and older adults without memory complaints.

**Method**: Data were obtained from the Alzheimer’s Disease Neuroimaging Initiative (ADNI) database from 19 participants with SCD and 19 similarly-aged controls. For each participant, a map of BOLD signal variability (SD_BOLD_) was computed as the standard deviation of the BOLD time-series at each voxel. Group comparisons were performed to examine differences in resting-state SD_BOLD_ in SCD vs. healthy controls. Relationships were then examined between participant SD_BOLD_ maps and neuroimaging markers of white matter vascular infarcts in each group separately.

**Results**: Between-group comparisons showed no significant differences in whole-brain SD_BOLD_ in individuals with SCD and controls. In the healthy aging group, higher white matter hyperintensity (WMH) burden was associated with greater SD_BOLD_ in right temporal regions (*p* < 0.05), and lower scores on a measure of global executive functioning. These associations were not identified in individuals with SCD.

**Conclusion**: The current study underscores previous evidence for a relationship between SD_BOLD_ and white matter vascular infarcts in the healthy aging brain. The findings also provide evidence for a dissociable relationship between healthy aging and SCD, such that in healthy controls, increased WMH is associated with declines in executive function that is not observed in older adults who present with memory complaints. Further multimodal work is needed to better understand the contributions of vascular pathology to the BOLD signal, and its potential relationship with pathological aging.

## Introduction

Dementia is one of the leading causes of disability and dependency in older adults worldwide (World Health Organization, 2017). Alzheimer’s Disease (AD) accounts for approximately 70% of all cases of dementia (Reitz et al., 2011). Although AD is a multifaceted disorder stemming from the complex interplay of both environmental and genetic risk factors, evidence continues to suggest that the strongest risk factor for AD is age. It is expected that by age 85, nearly one out of three individuals will develop AD (Alzheimer’s Association, 2016). With a now rapidly aging global population, the worldwide prevalence of AD is expected to reach 106 million by the year 2050 (Norton et al., 2014). Given this rapid rise in prevalence, even a small reduction in the incidence of AD is projected to have tremendous effects on its associated social and economic burden (Norton et al., 2014; Shatenstein et al., 2015).

Despite this urgent public health concern, there are no curative treatments for AD (Scheltens et al., 2016). Available treatment options are limited and focus primarily on delaying the progression of symptoms (Alzheimer’s Association, 2016). In order to be most effective, treatments are best administered at the earliest stages of the disease, when cognitive functioning is largely preserved (Rabin et al., 2017). In support of this goal, recent research on AD has turned to the identification of reliable, early biomarkers (Jack et al., 2010). While it is now well-established that the cognitive impairments in AD are preceded by several decades of asymptomatic neurodegenerative and vascular lesions, few reliable biomarkers of this pre-clinical phase exist (Jack et al., 2010; Shatenstein et al., 2015). Recently, advances in the AD literature have revealed that an individual’s *subjective* experience of cognitive decline may be one of the earliest symptomatic markers of the AD neurobiological cascade, even in the absence of any measurable cognitive impairment (Jessen, 2014).

In the current literature, the phenomenon of subjective cognitive decline (SCD) refers to a pre-clinical stage of AD in which an individual perceives a decline in their memory and/or other cognitive abilities, in the absence of any objective neuropsychological deficits (Jessen, 2014; Rabin et al., 2017). SCD is believed to precede the symptomatic pre-dementia phase of AD, known as mild cognitive impairment (MCI), during which cognitive deficits can be measured on standardized tests (Rabin et al., 2017). Specifically, SCD marks the *intermediary* stage between normal aging and MCI, where the threshold for impairment has yet to be reached, ostensibly due to successful compensation in the very early stages of the disease (Rabin et al., 2017).

As the earliest available indicator of the AD process, further studies in individuals with SCD have the potential to reveal important insights on the biological correlates of this critical treatment stage. Indeed, a number of studies have shown that SCD is associated with AD biomarkers across a range of imaging modalities (Rabin et al., 2017). For instance, some structural magnetic resonance imaging (MRI) studies have shown that, relative to older adults without memory complaints, individuals with SCD exhibit reductions in hippocampal volumes and atrophy in core medial and fronto-temporal regions (Jessen et al., 2006; Saykin et al., 2006; Meiberth et al., 2015; Perrotin et al., 2015). Recent studies have also demonstrated evidence of widespread reductions in white matter integrity in individuals with SCD (Ohlhauser et al., 2019). In contrast, some studies have failed to find structural changes in patients with SCD, but have reported differences in brain *function*, as measured by blood-oxygen-level-dependent functional MRI (BOLD fMRI). Notably, some neuroimaging findings in AD suggest that changes in brain function may actually *precede* changes in brain structure (Damoiseaux et al., 2012). For instance, a series of studies have found *increased* functional activation in temporal, frontal, and occipitoparietal regions during episodic memory encoding and divided attention tasks in patients with SCD (Rodda et al., 2009, 2011). Increased activation in a number of parieto-temporal and occipital regions have also been identified in resting-state fMRI studies (rsfMRI; Sun et al., 2016). Overall, these patterns of functional alterations are consistent with regions of *decreased* activation typically seen in AD patients, thus potentially providing neural support for the compensatory theory in SCD (Rodda et al., 2011; Sun et al., 2016). However, findings to date are mixed, with some studies showing increased and others decreased activity in both AD and SCD groups (Schwindt and Black, 2009; Erk et al., 2011). Therefore, few reliable fMRI biomarkers of AD or SCD have been identified.

Traditionally, the majority of these rsfMRI investigations have based their findings on patterns of *mean* brain activity. Recently, novel approaches to analyzing rsfMRI data have been developed that focus on the moment-to-moment variability in the BOLD signal (Garrett et al., 2013). In recent years, an increasing number of studies have focused on BOLD signal standard deviation (SD_BOLD_) in normative aging (Garrett et al., 2013). Many of these studies have found that patterns of resting-state SD_BOLD_ are generally *more* variable in younger adults, with less variability believed to reflect reductions in synaptic complexity with increasing age (Garrett et al., 2010, 2013). Other studies have also found *greater* resting-state SD_BOLD_ to be associated with increased integrity of white matter pathways in healthy older adults (Burzynska et al., 2015a,b). In contrast to these findings, a number of studies have also found regional *increases* in fMRI BOLD variance in older vs. younger healthy adults (Garrett et al., 2010, 2011; Nomi et al., 2017), as well in a number of patient populations including AD, stroke, multiple sclerosis, and neurogenetic disorders (Kielar et al., 2016; Makedonov et al., 2016; Petracca et al., 2017; Zöller et al., 2017; Scarapicchia et al., 2018).

Evidently, the source and nature of increased regional BOLD fluctuations in the literature remain unclear. Findings of BOLD variability in patient populations suggest that *increased* SD_BOLD_ may reflect sub-optimal functioning and/or compensatory mechanisms associated with age-related cognitive decline (Garrett et al., 2010; Nomi et al., 2017; Petracca et al., 2017; Scarapicchia et al., 2018). More recently, additional studies in older adults and patients with vascular risk factors [i.e., hypertension and cerebral small vessel disease (CSVD)] have found *greater* BOLD fluctuations in white matter regions relative to controls (Makedonov et al., 2013; Jahanian et al., 2014). These findings are believed to be indicative of increased arterial stiffness and pulsatility, suggesting that resting-state BOLD variance may actually serve as a physiological signal related to an individual’s cerebrovascular status (Makedonov et al., 2013; Jahanian et al., 2014).

Despite the close link between vascular impairment and cognitive decline, no studies to date have examined SD_BOLD_ in individuals with SCD. However, recent studies on BOLD fluctuations in patients with AD suggest that this novel analysis method may be able to provide valuable new insights into the disease process. For instance, a study by Makedonov et al. (2016) examined rsfMRI in white matter in individuals with AD and found significantly *increased* BOLD fluctuations in white matter in those with AD relative to MCI and age-matched controls. White matter BOLD fluctuations were also found to be negatively correlated with participant memory scores, supporting the notion that BOLD variability may be an indicator of neural compromise (Makedonov et al., 2016). More recently, the current group conducted a study using data from the Alzheimer’s Disease Neuroimaging Initiative (ADNI) to examine rsfMRI SD_BOLD_ in AD patients relative to healthy aging controls (Scarapicchia et al., 2018). In accordance with the findings by Makedonov et al. (2016), results from this study also revealed significantly *increased* BOLD fluctuations in patients with AD relative to healthy controls in right-lateralized frontal regions. In the normative aging group, it was further found that lower memory scores were associated with *greater* SD_BOLD_ in the medial temporal lobe and adjacent structures, supporting a tentative link between BOLD fluctuations and cognitive functioning (Scarapicchia et al., 2018). Based on previous studies suggesting that vascular factors may underlie BOLD fluctuations patterns in AD (Makedonov et al., 2013, 2016), we also examined associations between resting-state BOLD variability and a well-established MRI-based proxy of cerebrovascular status, namely: white matter lesion burden [white matter hyperintensities (WMH); Prins and Scheltens, 2015]. In accordance with the cognitive findings, an association was again identified in the healthy aging group only, showing a *positive* correlation between WMH lesion burden and SD_BOLD_. Further to this, greater vascular insult was uniformly associated with lower memory and executive functioning across the sample (Scarapicchia et al., 2018). Given the well-established association between vascular risk factors and AD, these findings suggest that SD_BOLD_ may provide important insights into cerebrovascular factors underlying cognitive aging and, potentially, the risk for AD.

The findings from our previous study are in line with ample evidence supporting an association between WMH volume and global cognitive impairment (Alber et al., 2019). Likewise, the association between vascular risk factors and AD is one that is well established (O’Brien and Markus, 2014; Cai et al., 2015). Although, in our previous study, the association between SD_BOLD_ and vascular burden was not identified in patients with AD, this is consistent with previous meta-analyses showing that WMHs tend to be associated with an increased risk of dementia and AD in aging populations, but *not* in patients who already exhibit cognitive impairment (Debette and Markus, 2010). Rather, it has been posited that the effect of WMH on cognitive decline may be most salient in the early stages of cognitive decline (Moon et al., 2017). To expand on our previous findings, it is clear that further exploration of the link between rsfMRI SD_BOLD_ and vascular factors are needed, particularly in pre-clinical groups, such as SCD.

Few studies to date have examined the relationship between WMH and cognitive decline in individuals with SCD, and none have examined the effects of vascular burden on fMRI measures of BOLD variability in this group. Existing studies in SCD suggest that increased WMH burden is associated with annual declines in processing speed, memory, executive functioning, and global cognition (van der Flier et al., 2005; Benedictus et al., 2015; Moon et al., 2017). Further to simply being a marker for underlying age-related vascular disease, WMHs are also thought to be related to vascular and non-vascular processes associated with the AD neurobiological cascade (Alber et al., 2019). For instance, a large seminal study found that young adults carrying dominant AD mutations had elevated WMH volumes 20 years prior to cognitive symptom onset, suggesting that WMHs may actually be a *pathogenic* component of the disease (Lee et al., 2016).

In light of the evidence reviewed, examining SD_BOLD_ as a non-invasive biomarker for underlying cerebrovascular status has the potential to support our ultimate goal of improving the identification of AD in its pre-clinical stages. By applying this novel approach to the *earliest* symptomatic phase (SCD), we can hopefully obtain novel insights into the neurophysiological correlates of the AD cascade and identify potential therapeutic targets. To this end, the current study aims to: (1) examine whole-brain differences in SD_BOLD_ in a group of individuals with SCD compared to elderly controls; and (2) investigate the relations between BOLD signal variability and WMH burden. Based on the findings from our previous study, we expect to find: (1) increased rsfMRI BOLD variances in patients with SCD vs. healthy controls; and (2) a positive relation between BOLD variability and MRI-based measures of the cerebrovascular infarct.

## Materials and Methods

### ADNI Database

All data for the present study were obtained from the ADNI-2 database^1^. The ADNI, led by principal investigator Michael W. Weiner, began in 2003 as a partnership between the National Institute on Aging, the National Institute of Biomedical Imaging and Engineering, the Food and Drug Administration, as well as other private and public non-profit organizations. All data from the ADNI has been collected from acquisition sites across Canada and the United States according to the standardized ADNI protocol. The initial goal of ADNI was to develop sensitive methods that would be able to detect AD at its earliest time point, in order to maximize the efficacy of future disease-modifying or delaying interventions. With the later inclusion of the “ADNI-Grand Opportunities” (ADNI-GO) and ADNI-2 phases, the project has since expanded to examine biomarkers of the *pre-dementia* stage through the addition of preclinical groups such as SCD and amnestic MCI. The ADNI-3 is focused on understanding the progression of AD, and the interplay between imaging, laboratory, and neuropsychological AD biomarkers over time. For further information, please see http://www.adni-info.org.

### Participants

All participants were selected from the ADNI-2 database. In this database, participants with SCD are categorized as having significant memory complaints (SMC) and labeled as such in the protocol. For the purpose of continuity with the existing literature, the current study will refer to this group as “SCD,” given the conceptual equivalence of the two labels.

In order to ensure the best variable control possible, inclusion in the study required participants to have: (1) a clinician-confirmed diagnosis of SMC or “normal” at the screening visit; and (2) complete rsfMRI data available for the first time point of the study. Given that ADNI participants are classified into groups based on their clinical presentation at the screening visit, SCD participants were selected from the first available time point (approximately 14 days post-screening) to ensure the continued accuracy of this diagnosis. An equivalent number of control participants were then selected to form a comparable group. In the end, data were obtained from the first available time point from 19 individuals with SCD (mean age = 72.2 years, SD = 5.2; 10 females) and 19 healthy aging controls (mean age = 74.7, years, SD = 6.9; 11 females). Participant demographic information can be found in Table 1.

**Table 1:** Participant demographics.

	SCD	CN
Age	72.2 ± 5.2	74.7 ± 6.9
Females	10	11
Males	9	8
Education (years)	16.3 ± 3.16	16.3 ± 2.3

Group classification of SCD participants was made by ADNI investigators. Participants in this group were self-referrals who presented with a significant *subjective* memory concern, confirmed by a score ≥16 on the first 12 items of the Cognitive Change Index questionnaire (CCI; Saykin et al., 2006). In addition, participants in this group were required to have normal memory function on the Logical Memory II subscale of the revised WMS (WMS II, ≥9 for 16 years of education and above), a Mini-Mental State Exam (MMSE) score between 24 and 30 (inclusive), and a Clinical Dementia Rating of 0, with the Memory Box score, also marked as 0. Aside from subjective complaints, participants in this group were cognitively normal based on the absence of objective cognitive impairments in memory or activities of daily living.

All control participants were free of subjective memory complaints and deemed cognitively normal based on clinical assessments by the site physician showing an absence of significant impairment in cognitive functioning and performance of daily activities. Similar to the SCD group, participants in the control cohort exhibited normal memory function on the Logical Memory II subscale of the revised WMS (WMS II, ≥9 for 16 years of education and above), an MMSE score between 24 and 30 (inclusive), and a Clinical Dementia Rating of 0. For more information on group classifications, including all additional eligibility criteria, please consult the ADNI-2 procedures manual (Alzheimer’s Disease Neuroimaging Initiative, 2008).

All ADNI participant’s written informed consent approved by the Institutional Review Board at each acquisition site. Secondary use of the data was approved by the Human Research Ethics Board at the University of Victoria (British Columbia, Canada).

### Image Acquisition

MRI data were downloaded with permission from the ADNI. Based standardized site protocols, all images were acquired on 3.0 Tesla Philips MRI scanners across 10 North American acquisition sites (Jack et al., 2008). Specifically, whole-brain anatomical MRI scans were acquired sagittally, with a T1-weighted MPRAGE sequence, with the following parameters: 1.2 mm slice thickness, 256 × 256 × 170 acquisition matrix, echo time (TE) of 3 ms, in-plane voxel dimension of 1 mm^2^, repetition time (TR) of 7 ms, and flip angle of 9°. fMRI scans were obtained during resting state, in which participants were instructed to lay quietly in the scanner with their eyes open (scan duration ≈10 min). Resting-state fMRI scans were obtained with a T2*-weighted echo-planar imaging sequence with the following parameters: 140 volumes, 64 × 64 × 48 acquisition matrix (voxel size = 3.3 mm^3^), TE of 30 ms, TR of 3,000 ms, and flip angle of 80°.

The participant’s T2-weighted fluid-attenuated inversion recovery (FLAIR) images were obtained for the purpose of lesion volume computation (WHM burden). The T2-weighted FLAIR images were obtained with 5.0 mm axial slices, a 256 × 256 × 35 acquisition matrix, a TE of 90 ms, an inversion time of 2,500 ms, an in-plane voxel dimension of 0.85938 mm^2^, a TR of 9,000 ms, and a flip angle of 90°.

### Data Analyses

In order to ensure methodological continuity between studies and facilitate the comparison of findings, the following methods are based on the neuroimaging processing and analysis protocols outlined in Scarapicchia et al. (2018).

#### Image Preprocessing

All analysis steps were performed using tools within the fMRI of the Brain Software Library (FSL) version 5.0 (Analysis Group, FMRIB, Oxford, UK^2^; Smith et al., 2004). Non-brain tissue in the raw T1 images was removed using the automated Brain Extraction Tool (BET; Smith, 2002), followed by manual verification and optimization for each subject. BOLD data preprocessing was performed in FSL’s FEAT as follows: each functional (T2*) image was processed with a highpass temporal filter (125 s) and the non-brain signal was removed using FSL’s functional BET. The functional images were subsequently motion-corrected and registered to their high-resolution T1 structural image that was linearly registered to standard stereotaxic space using 12 degrees of freedom transformation. A nonlinear registration (10 mm warp resolution) of the structural image to standard stereotactic space was also applied to account for potential local deformations in brains of the patient group. In accordance with previous groups, global signal regression (GSR) was performed to correct for confounding physiological noise and to improve the detection of localized variation in the BOLD signal (Desjardins et al., 2001; Macey et al., 2004; Fox et al., 2009).

#### Statistical Comparisons

Individuals with SCD were compared to healthy aging controls in the group-level analysis. Associations between BOLD variability and WMH burden were examined separately for each group to reduce the likelihood of Type I error due to random population variance. All statistical models were computed using FSL’s General Linear Model, whereby the effect at each voxel is modeled as a linear combination of one or more predictors. All results were examined at a *p* < 0.05 (corrected) significance level unless otherwise stated.

#### Resting-State BOLD Variability (SD_BOLD_)

Though different conceptualizations of brain signal variability exist, a commonly used approach in rsfMRI analysis involves examining the distributional width of the neuroimaging time-series by computing the signal variance or standard deviation (SD_BOLD_) within voxels across the brain (for a review of alternate BOLD variability measures, see Garrett et al., 2013). In line with the approach in our previous study, we derived a measure of BOLD variability by first obtaining the variance of the residual signal leftover after preprocessing at each voxel across the whole brain, in both gray and white matter tissues collectively This whole-brain approach is in keeping with an increasing number of studies suggesting that important fMRI signal activation can be found in white matter regions, given the many vascular innervations in these structures (e.g., Gawryluk et al., 2014; Grajauskas et al., 2019). The square root of the variance within each voxel was then subsequently computed in order to arrive at an SD_BOLD_ map for each participant. The derived SD_BOLD_ maps from each individual were then merged into a single 4D file and smoothed with a Gaussian kernel (6 mm). To examine differences in resting-state BOLD variability in participants with SCD vs. healthy age-matched controls, between-group contrast comparisons of SD_BOLD_ were performed using randomize (Winkler et al., 2014) with threshold-free cluster enhancement and correction for multiple comparisons. While it is plausible that differences in brain atrophy across groups may impact whole-brain SD_BOLD_ maps, previous morphometric comparisons of the same groups have failed to find significant volumetric differences (Parker, 2019), thereby accounting for any partial volume effects.

#### Relations Between BOLD Variability and Cerebrovascular Status (WM Lesion Volumes)

In light of the evidence reviewed that links WMHs to pathogenic AD processes, in the current study, neuroimaging markers of vascular burden were used as a proxy for an individual’s cerebrovascular status. Specifically, we identified participant WMHs on FLAIR images and determined total WM lesion burden for each participant. Lesions were segmented by the Lesion Prediction Algorithm (LPA; Schmidt, 2017), using FLAIR images only, as implemented in the LST toolbox version 2.0.15^3^ for the Statistical Parametric Mapping (SPM) software. The LPA is a binary classifier in the form of a logistic regression model that was initially trained on the data of multiple sclerosis patients with severe lesion patterns (Schmidt et al., 2012). Parameters of this model are used to segment lesions in new images by computing an estimate for the lesion probability within each voxel. Owing to the Bayesian inference methods used in the LPA, threshold parameters are thus computed internally and not subject to manual adjustment. In the current study, lesion volumes (ml) derived from each participant’s probabilistic map were extracted to derive a total lesion volume for each subject. To account for variability in parenchymal volume across participants, total brain volumes for each participant were computed using FMRIB’s Automated Segmentation Tool (FAST; Zhang et al., 2001) and followed by visual inspection of the outcome of brain volume. Lesion volumes were subsequently converted to a value representing the fraction of total brain volume occupied by WMHs in each participant (fractional lesion volume). Correlations were then examined between these values and the participants’ resting-state SD_BOLD_ maps. As in previous steps, statistical computations were performed using randomization with threshold-free cluster enhancement and correction for multiple comparisons.

#### Relations Between WMH Burden and Cognitive Performance

As a secondary *post hoc* analysis, the relation between cognitive performance and WMH burden was examined within each group to better understand the contributions of the cerebrovascular burden to cognitive performance within each sample. Specifically, associations were examined between WMH burden and composite clinical measures for: (1) memory performance (ADNI-MEM); and (2) executive function (ADNI-EF). Both of these cognitive measures have been derived from the ADNI neuropsychological test battery using item response theory methods and validated in subsequent studies. Specifically, the ADNI-MEM score was derived from a single-factor confirmatory factor analysis model performed by Crane et al. (2012) using data from the Rey Auditory Verbal Learning Test (RAVLT), AD Assessment Schedule—Cognition (ADAS-Cog), MMSE, and the WMS Logical Memory subscale. The ADNI-EF score was derived by Gibbons et al. (2012) using a bi-factor confirmatory factor analysis model with data from the Wechsler Adult Intelligence Scale-Revised (WAIS-R) Digit Symbol Substitution, Digit Span Backwards, Trails A and B, Category Fluency, and Clock Drawing. Both composite scores are available in ADNI as a standardized metric with a population mean of 0 and a standard deviation of 1. Partial correlation coefficients were examined with two-tailed significance (*p* < 0.05), controlling for both age and education.

## Results

### Differences in Resting-State SD_BOLD_ in Patients With SCD vs. Healthy Controls

**Figure 1:**
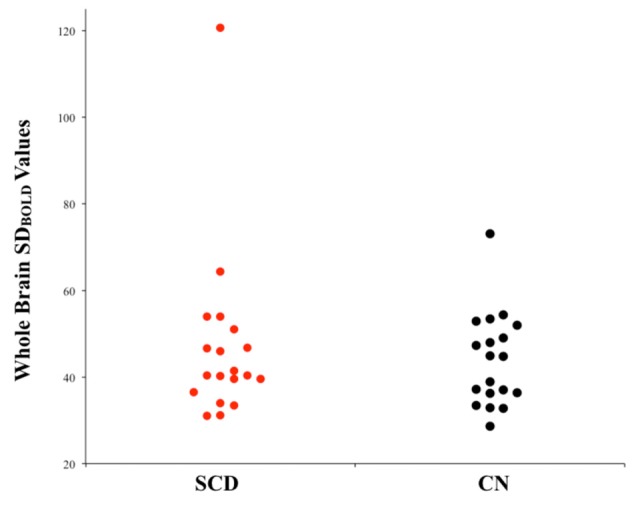
Univariate scatterplot demonstrating the lack of significant difference in blood-oxygen-level-dependent (BOLD) variability between individuals with subjective cognitive decline (SCD) and controls. Plotted values within each group represent whole-brain SD_BOLD_ values for each participant.

Between-group comparisons were performed to examine differences in resting-state SD_BOLD_ in participants who present with SCD relative to healthy aging participants without memory complaints. Contrasts were examined bidirectionally (i.e., SCD > CN and SCD < CN). Following standard statistical thresholding (*p* < 0.05) and corrections for multiple comparisons, the results of this initial analysis did not reveal significant differences in whole-brain resting-state BOLD variability between the two groups, in white or gray matter (Figure 1).

### Relations Between Resting-State SD_BOLD_ and Participant WMH Volume

**Figure 2:**
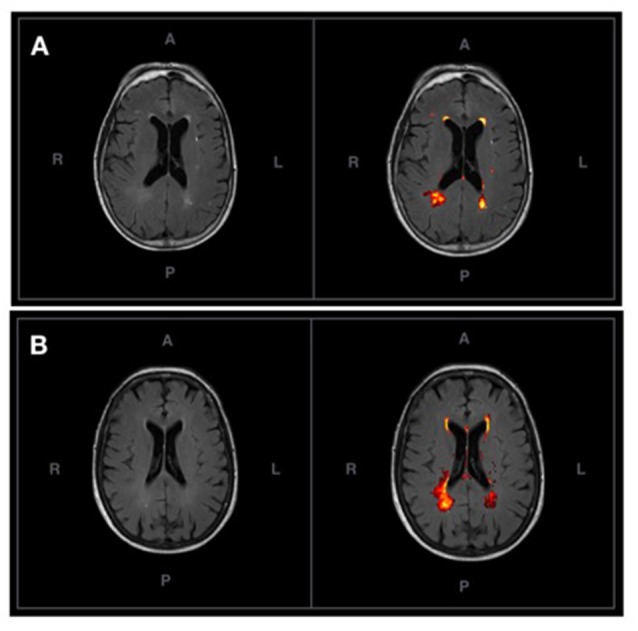
**(A)** An axial T2-FLAIR image of a prototypical participant from the control group (left) and associated probabilistic lesion volume map (right) generated by the LST-LPA. **(B)** An axial T2-FLAIR image of a prototypical participant with SCD (left) and associated probabilistic lesion volume map (right) generated by the LST-LPA. Prototypical SCD and healthy control participants were selected based on the proximity of their white matter (WM) lesion burden to their respective group means.

**Figure 3:**
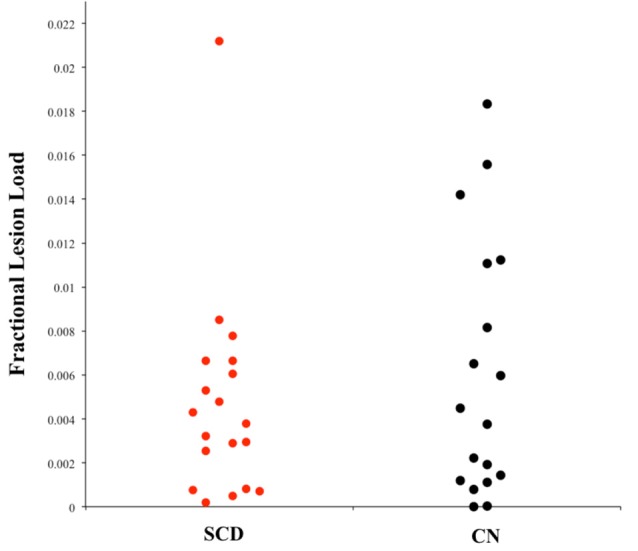
Univariate scatterplot demonstrating the dispersion of fractional lesion load values in each group. Note the restriction of range in the SCD group that is not apparent in individuals without memory complaints (CN).

Figure 2 shows the probabilistic lesion volume maps for a representative SCD and healthy control participant. To account for total brain volume, lesion volumes obtained from the lesion segmentation tool were subsequently converted to a value representing the fraction of total brain volume occupied by WMH in each participant (fractional lesion load). Overall, the two groups did not differ significantly in WMH burden. Further examination of the sample revealed an outlier in the SCD group, which presented with a significantly higher fractional lesion load than the group mean (0.02). While removing the outlier results in a marginally higher mean fractional lesion load in the SCD group (0.0037 ± 0.0025), there remains no statistically significant difference in WMH burden between the two groups. Additional descriptives of WMH burden in each group can be found in Table 2, Figure 3.

**Table 2:** White matter lesion burden: group characteristics.

	SCD*	CN*	SCD vs. CN
Raw lesion volume (cm^3^)	5.1 ± 6.0	7.3 ± 7.7	*p* = 0.3
Brain volume (cm^3^)	1,051.1 ± 145.2	1,029.5 ± 110.7	*p* = 0.6
Fractional lesion load	0.0047 ± 0.0047	0.0069 ± 0.0069	*p* = 0.3

*Values listed represent mean ± SD.

**Figure 4:**
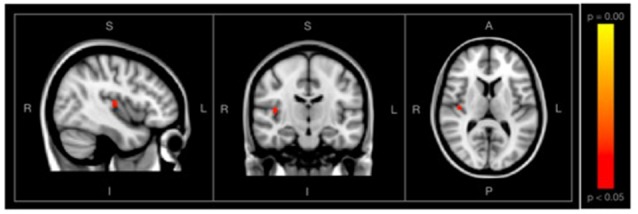
Images showing regions where SD_BOLD_ is positively associated with WM hyperintensity (WMH) burden in the healthy control group, shown with conventional statistical thresholds (*p* < 0.05, corrected for multiple comparisons). Images on overlaid on T1-weighted MNI152_T1_2mm standard template provided by the functional magnetic resonance imaging (fMRI) of the Brain’s Software Library.

Using the fractional lesion load values described, associations between WMH burden and SD_BOLD_ maps were examined separately within each group. Using a conservative significance threshold (*p* < 0.05) no significant relationship was found between total WMH burden and SD_BOLD_ in participants with SCD. However, a relationship was identified in the healthy control group, which showed a positive association between WMH lesion burden and SD_BOLD_, predominantly in the right transverse temporal gyrus, as well as portions of the right insula (Figure 4).

### Relations Between WMH Burden and Cognitive Performance Within Groups

**Figure 5:**
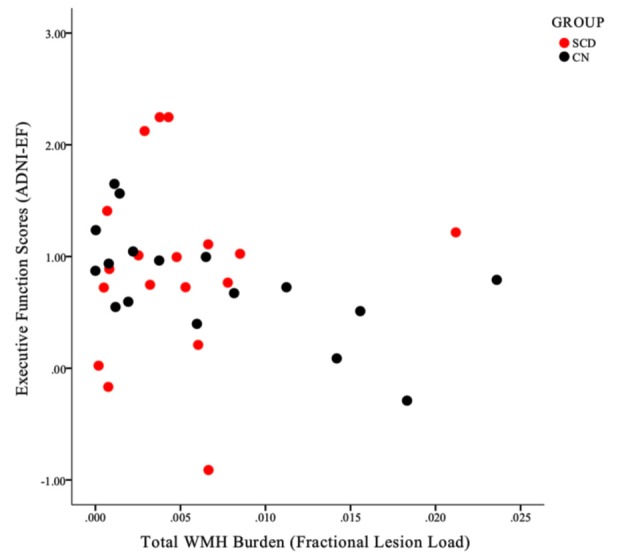
A scatterplot illustrating the differential relationship between ADNI-EF scores and total WMH burden within the SCD (red circles) and control (black circles) groups. Note that for individuals in the CN group, a clear negative linear association can be identified, such that increased WMH burden is associated with lower ADNI-EF scores within individuals. This linearity is not apparent in the SCD group.

As expected, based on the group selection criteria, the SCD and healthy control groups did not differ significantly on summary scores of memory (ADNI-MEM) or executive function (ADNI-EF; Table 3). With respect to the association between cognitive scores and vascular burden, a significant negative correlation (*r* = −0.601, *p* < 0.05) was found between ADNI-EF and WHM burden in the healthy control participants, after correcting for age and education. In contrast, no association was found between ADNI-EF scores and total WMH burden in the SCD group. This differential relationship is further illustrated in Figure 5. Finally, no significant association was found between ADNI-MEM and WHM in either group (Table 4).

**Table 3:** Summary scores of memory and executive function: group characteristics.

	SCD (*n* = 18*)	CN (*n* = 17**)	SCD vs. CN
ADNI-MEM^†^ (mean ± SD)	0.9351 ± 0.5174	0.8265 ± 0.49986	*p* = 0.533
ADNI-EF^†^ (mean ± SD)	0.9101 ± 0.81636	0.7824 ± 0.48144	*p* = 0.580

^†^Mean-centered ADNI composite scores (mean = 0 ± SD 1). *One participant omitted due to missing data. **Two participants omitted due to missing data.

**Table 4:** Partial correlation coefficients (*r*) for the relation between cognitive performance (ADNI-MEM and ADNI-EF) and WMH burden within groups, corrected for age and education.

	Group	WMH Burden Corr. (*r*)	Significance (*p*)
ADNI-MEM	SCD	0.016	0.953
	Controls	−0.249	0.370
ADNI-EF	SCD	−0.042	0.876
	Controls	−0.601	0.018*

*p < 0.05

## Discussion

### Resting-State SD_BOLD_ in Participants With SCD Compared to Aging Controls

The first aim of the current study was to examine and characterize differences in resting-state BOLD variability in individuals with SCD relative to aging participants without memory complaints. Initial studies on BOLD variability have reported robust associations between regional BOLD variability and age-related cognitive decline (Garrett et al., 2010, 2011, 2013). Most recently, a previous study by the current group found significant increases in SD_BOLD_ in patients with Alzheimer’s Disease relative to healthy aging controls in a number of right-lateralized gray and white matter regions (Scarapicchia et al., 2018). Based on these findings, which together suggest that increased SD_BOLD_ may serve as a marker for sub-optimal brain functioning, we anticipated that individuals with SCD would present with increased SD_BOLD_ relative to aging controls without memory complaints. Contrary to our hypothesis, whole-brain analyses between SCD and controls ultimately did not reveal any significant differences in SD_BOLD_ between the two groups, in either gray or white matter regions.

There are a number of potential explanations for the above findings, many of which are contingent upon theoretical and methodological interpretations of the rsfMRI SD_BOLD_ signal. As previously discussed, there is a lack of consensus on the source and nature of BOLD fluctuations in the current literature. Neural theories of BOLD variability suggest that changes in SD_BOLD_ could reflect sub-optimal functioning and/or compensatory mechanisms associated with age-related cognitive decline, or even reductions in synaptic complexity (Garrett et al., 2010, 2013; Nomi et al., 2017; Petracca et al., 2017; Scarapicchia et al., 2018). Interpreted within the context of this framework, the current finding of no significant difference in SD_BOLD_ between participants with and without SCD can be seen as congruent with the absence of objective cognitive differences between these two groups, with the sole exception of self-reported memory complaints. To this end, Makedonov et al. (2016) also failed to find a significant difference in white-matter BOLD variability between healthy aging and MCI participants from the ADNI database, thereby supporting what appears to be a dampening of significant effects further along the AD disease continuum. This stands in contrast to the widespread neurological differences commonly observed between AD and healthy aging, between which differences in rsfMRI SD_BOLD_ have been identified in previous studies (Makedonov et al., 2016; Scarapicchia et al., 2018).

While some functional brain changes have previously been identified in individuals with SCD, it is notable that the overwhelming majority of these findings have reflected functional compensation during *active* tasks of cognition, rather than resting-state (Mizuno et al., 2018). Moreover, in resting-state studies of SCD that have been conducted, all have examined conventional *mean-based* patterns of activation. While related mean- and variability-based measures of brain activity are related, the results of each analysis are statistically orthogonal parameters which have been suggested to reflect different aspects of brain function not otherwise captured by conventional fMRI (Garrett et al., 2010, 2011). It is therefore plausible that individuals with SCD and healthy aging controls do not differ on these novel parameters, which, in neural theories, are purported to reflect synaptic integration and complexity, rather than a clear compensatory over-recruitment of affected regions.

Conversely, other theories in the literature purport that rsfMRI BOLD fluctuations may reflect underlying changes in cerebrovascular integrity, that which itself may be further associated with increased independent risk for AD (Makedonov et al., 2013, 2016; Jahanian et al., 2014; DeVis et al., 2018). In such interpretations, a finding of a lack of regional variation in SD_BOLD_ between participants with SCD and controls may reasonably reflect (i) a lack of significant difference in vascular anomalies between the two groups and/or (ii) dissociable relationships between SD_BOLD_ and vascular factors within each group. These queries are further explored in our within-group analyses, described below.

### SD_BOLD_ and its Association With Global WMH Burden Within-Groups

Following these initial comparisons, a second aim of the current study was to quantify and examine vascular contributions to the BOLD signal, in order to examine its effects on measures of whole-brain SD_BOLD_ in each group. Based on existing vascular theories of SD_BOLD_, we expected to find a positive relationship between BOLD variability and MRI-based measures of cerebrovascular infarct within each group. Final WMH volumes were derived from automated probabilistic mapping and were subsequently corrected for total parenchymal volume within each participant. Overall, the two groups did not differ in mean WMH burden (Table 2). To examine the effect of WMH burden on rsfMRI SD_BOLD_, associations between fractional lesion volumes and SD_BOLD_ maps were examined within each group, separately. Although no associations between whole-brain SD_BOLD_ and WMH burden were identified in the SCD group, a *higher* WMH burden was significantly (*p* < 0.05, corrected) associated with *increased* rsfMRI SD_BOLD_ in the control group, in right-lateralized regions of the insula (Figure 4). This finding is commensurate with our previous trend-level results in the same group, which found a positive trend-level association between increased SD_BOLD_ and WMH in healthy controls in widespread temporal regions, with no association in patients with AD (Scarapicchia et al., 2018). Given that this series of studies are among the first to examine this relationship, additional work is needed to better understand the regional significance of these findings, as well as the possibility that they may be related to underlying vasculature in these regions, rather than cortical gray matter *per se*.

Specifically, in keeping with neurovascular theories of SD_BOLD_, it is possible that BOLD variability observed in healthy aging may represent the physiological consequences of white matter lesion accumulation and associated regional vascular rigidity. Although similar to our previous findings, the current results serve to underscore our previous analyses in this same group, due to refinements in the LST-SPM toolbox algorithm that have allowed for increased sensitivity and specificity of white matter lesion segmentation. Altogether, these findings reaffirm the association between WMH burden and rsfMRI SD_BOLD_ in healthy aging, specifically in the absence of objective *and* subjective memory impairments. In keeping with theories purporting that SCD represents the earliest symptomatic phase of the AD neurobiological cascade, one plausible explanation is that this linear association is one that is progressively lost in later phases of the disease, perhaps beginning as early as SCD and becoming more pronounced in the AD stage. Indeed, previous reviews on WMH in older adults have estimated that the rate of WMH-related functional degeneration is approximately twelve times lower than the rate of any degeneration attributed to AD-specific processes (Frisoni et al., 2007). It is possible, then, that this loss of association between WMH and BOLD variability may represent the beginning stages of more advanced structural or functional brain aberrations that may overpower any effect that can otherwise be observed in the healthy aging brain. Clinically, this finding may represent a potential physiological marker of neural compromise, in the absence of psychometric evidence. Our additional finding of a dissociable relationship between WMH and cognitive function in each group discussed further below, lends further support to this theory.

### Impact of Global Cerebrovascular Infarct on Executive Function and Memory

In order to further explore the contributions of WMH burden to cognitive impairment in each group, *post hoc* analyses examined the association between white matter lesion volumes and composite scores of executive function (ADNI-EF) and memory (ADNI-MEM). In line with our within-group SB_BOLD_ findings, a dissociable relationship emerged between the two groups, whereby a significant correlation between WMH burden and cognition was identified in the healthy control group, but *not* in those with SCD (Figure 5). Specifically, it was found that a higher total WMH burden was associated with poorer global executive function (*r* = −0.60) in control participants who did not present with subjective memory complaints. Notably, this association was not identified at conservative or trend-level thresholds for the SCD group. Interestingly, this association was also not identified with respect to memory performance scores in either group, despite the common clinical focus on memory performance as a central feature of the AD neurobiological cascade.

Taken together, the results outlined in the current article, as well as in our previous study, provide evidence for a relationship between rsfMRI SD_BOLD_ and white matter vascular infarcts in the healthy aging brain. Novel to the current study is the finding that these effects appear to dissipate in the presence of overt (AD) and subjective (SCD) neural compromise. Notably, in healthy aging groups where this association is tenable, there furthermore appears to be a correlation between WMH burden and declines in global executive function that is not seen in participants with SCD (Figure 5). In keeping with these results, it is plausible that SD_BOLD_ may represent a normative physiological marker of cerebrovascular compromise in *healthy* aging brains. Following from this, it is also logical that such vascular aberrations, when present, would then be associated with declines in global executive functioning. Indeed, it has been demonstrated that, regardless of their localization, white matter lesions are uniformly associated with frontal hypometabolism and executive dysfunction, with the strongest effect sizes often observed in groups who do *not* present with cognitive impairment or dementia (Tullberg et al., 2004).

More broadly, the link between WMHs and executive dysfunction is one that has seen increasing evidence in recent years (Hedden et al., 2012; Arvanitakis et al., 2016; Boyle et al., 2016). While WMH burden has also been linked to progressive declines in episodic memory and pathophysiological markers of AD (O’Brien and Markus, 2014), other large-scale studies have found that the association between WMH burden and episodic memory is almost entirely mediated by the impact of vascular burden on executive functioning (Parks et al., 2011). To that end, it is notable that in the current study, individuals who presented with subjective memory complaints did *not* exhibit an association between WMH burden and scores of executive functioning. Future longitudinal work examining the link between executive function, memory, and cerebrovascular burden in individuals with SCD may aid in clarifying these dissociable findings in this group, particularly with multiple measures that capture specific components of executive function (e.g., planning, inhibition, self-monitoring).

### Limitations and Future Directions

A primary limitation of fMRI BOLD variability studies as a whole relates to the many conceptualizations of “BOLD variability” that exist in the current literature. While the present study has reviewed some of the challenges associated with interpreting fluctuations in the BOLD signal, methodological conceptualizations have also varied considerably across studies. To date, several different variations of “BOLD variability” measures have been described, with a considerable range in the methodology used to derive them (e.g., amplitude, variance, standard deviation, mean squared successive difference; for a review, see Garrett et al., 2013). This has greatly limited the ability to make systematic comparisons across studies. In an effort to improve upon this limitation, the current study followed the methods outlined in Scarapicchia et al. (2018) to allow for contiguity and comparison between these two studies on age-related pathologies. Increased efforts towards methodological standardization in future studies with different clinical samples would greatly aid in understanding the association between BOLD signal fluctuations and neurological disorders. Further, incorporating alternative neuroimaging methods with superior temporal properties, such as functional near-infrared spectroscopy, would also allow for a more precise understanding of the neurovascular properties that contribute to fluctuations in the BOLD signal.

In addition to challenges in systematically capturing “BOLD variability,” there also exists considerable debate in the literature regarding the measurement and identification of vascular lesions in structural neuroimaging. Despite its increasing clinical relevance in cerebrovascular research, recent systematic reviews have revealed a considerable heterogeneity in the methods used to define and segment vascular lesions (Frey et al., 2019). Consistent with the majority of published neuroimaging studies in this area, the current study utilized an automated segmentation tool to quantify hyperintense lesions on T2-FLAIR images (Schmidt et al., 2012; Schmidt, 2017). However, while WMHs are widely considered an imaging marker of CSVD (Mok and Kim, 2015) and cerebral arterial stiffness has found to be correlated with WHM lesion volume (Kidwell et al., 2001), WMHs are inherently limited as a proxy of total vascular status. Other lesions including lacunes and subcortical microinfarcts also figure prominently in CSVD, despite challenges capturing these infarcts with conventionally available segmentation tools (Wardlaw et al., 2013). More recently, there has also been a debate on the potential clinical relevance of differentiating periventricular vs. subcortical WMHs (Wardlaw et al., 2013; Kandel et al., 2016). Future investigations would likely benefit from the use of multimodal MRI and other imaging methods to fully explore the contributions of vascular lesions to the BOLD signal.

Though the current results do not appear to support a significant difference in rsfMRI BOLD variability between individuals with SCD and controls, further considerations are warranted. All participants from the current study were obtained from the ADNI database, which classifies individuals with SCD based on the severity of their self-reported subjective memory complaints (SMC). As SCD is still fairly new as a classification term, many approaches have been used to operationalize and quantify the construct, with little overlap among existing studies (Rabin et al., 2015). Classifying groups based on self-reported memory complaints is the most common approach taken in the literature on SCD, accounting for approximately 60% of studies (Rabin et al., 2015). However, it has been suggested that SCD may also encompass cognitive complaints outside of memory, such as executive function and attention, which were not included in the current SCD group (Reisberg and Gauthier, 2008). Indeed, newly published recommendations from the SCD Initiative (SCD-I) Working Group suggest that multiple cognitive domains, and particularly executive functions, be included in SCD criteria for research settings (Molinuevo et al., 2017). Finally, it is also possible that individuals with SCD recruited from community settings may differ from SCD populations recruited in clinic settings, the latter of which is the primary method of recruitment in the ADNI database.

Other sample considerations can also be made with respect to the null findings of an association between rsfMRI BOLD variability and vascular burden in SCD. Currently, participants in the ADNI database are required to be free of other major neurological disorders, psychiatric comorbidities, and significant vascular concerns, reflecting a highly sanitized sample characteristic of clinical trial populations (Petersen et al., 2010). Perhaps owing to these strict exclusion criteria, the WMH burden in the ADNI database is far less variable than that observed in other large-scale neuroimaging studies on aging (see Ramirez et al., 2016). Thus, the WMH burden observed in the current study is likely lower than what might be expected in a general population with subjective memory complaints. This may have contributed to underpowered effects. Moreover, in contrast to our findings of equal WMH volumes, studies in other groups have found that individuals with SCD typically present with significantly* greater* WMH burden than healthy elderly without SCD (e.g., van Rooden et al., 2018). It is strongly recommended that future studies employ more lenient inclusion criteria for vascular pathologies in their samples, thereby allowing for a more ecologically valid representation of the relationship between SCD, vascular burden, and the utility of rsfMRI BOLD fluctuations as a clinical biomarker of the former.

Finally, the sample size of the current study is small and limited to a single time point. To acquire a better understanding of BOLD variability and its association with cognition and vascular pathology over time, future studies in healthy aging will require larger samples and multiple age cohorts. Ideally, these cohorts would span the period of middle to late adulthood, when vascular pathologies and symptomatic complaints, respectively, are first believed to arise.

## Conclusion

AD is one of the leading causes of disability in older adults worldwide. A lack of curative treatments and a rapidly aging global population have rendered critical the need to identify early biomarkers of the disease process. In support of this goal, the current study employed novel rsfMRI techniques (SD_BOLD_) to examine brain signal changes in individuals with SCD vs. healthy aging. The findings outlined in the current article underscore previous evidence for a relationship between rsfMRI SD_BOLD_ and white matter vascular infarcts in the healthy aging brain. These effects appear to dissipate in the presence of subjective (SCD) and objective (AD) neural compromise, which may potentially represent an early physiological marker of neural compromise. Most significantly, the current study has demonstrated evidence for a dissociable relationship between groups, such that in healthy aging, a correlation is also found between increased WMH burden and declines in global executive function that is *not* seen in older adults who present with memory complaints. Further multimodal work in large healthy aging populations is needed to better understand the contributions of vascular pathology to the rsfMRI BOLD signal, and its potential relationship with cognitive decline over time.

## Data Availability Statement

Publicly available datasets were analyzed in this study. This data can be found here: Alzheimer’s Disease Neuroimaging Initiative Database.

## Ethics Statement

The studies involving human participants were reviewed and approved by University of Victoria Human Research Ethics Board. Written informed consent for participation was not required for this study in accordance with the national legislation and the institutional requirements.

## Author Contributions

The study was conceptualized by VS and JG. VS was responsible for data analyses and forming the initial draft of the manuscript. VS, JG, MG-B and SM were all involved in interpretation of the results, devising figures and tables and critically revising the manuscript.

## Conflict of Interest

The authors declare that the research was conducted in the absence of any commercial or financial relationships that could be construed as a potential conflict of interest.

## Footnotes

^1^http://adni.loni.usc.edu

^2^http://fsl.fmrib.ox.ac.uk

^3^http://www.statistical-modelling.de/lst.html

## Abbreviations

AD: Alzheimer’s disease
ADNI: Alzheimer’s Disease Neuroimaging Initiative
BOLD: Blood Oxygen-Level Dependent
FSL: Functional MRI of the Brain Software Library
MRI: magnetic resonance imaging
MMSE: Mini-Mental Status Exam
SD_BOLD_: Standard deviation of the fMRI BOLD signal
SCD: subjective cognitive decline
WMH: White matter hyperintensity
WMS: Wechsler Memory Scale.
